# Differentiation between Angiomatous Meningioma and Solitary Fibrous Tumors

**DOI:** 10.5334/jbsr.2759

**Published:** 2022-05-09

**Authors:** Martijn Verdam, Gert-Jan Allemeersch, Frans Van den Bergh

**Affiliations:** 1 Uz Brussel, BE

**Keywords:** Angiomatous Meningioma, Solitary Fibrous Tumor, Hemangiopericytoma, intralesional calcifications, early draining veins

## Abstract

**Teaching point:** The presence of intralesional calcifications, a dural tail sign, adjacent hyperostosis, and early draining veins can help distinguish angiomatous meningiomas (AM) from solitary fibrous tumors (SFT).

## Case History

**Figure 1:**
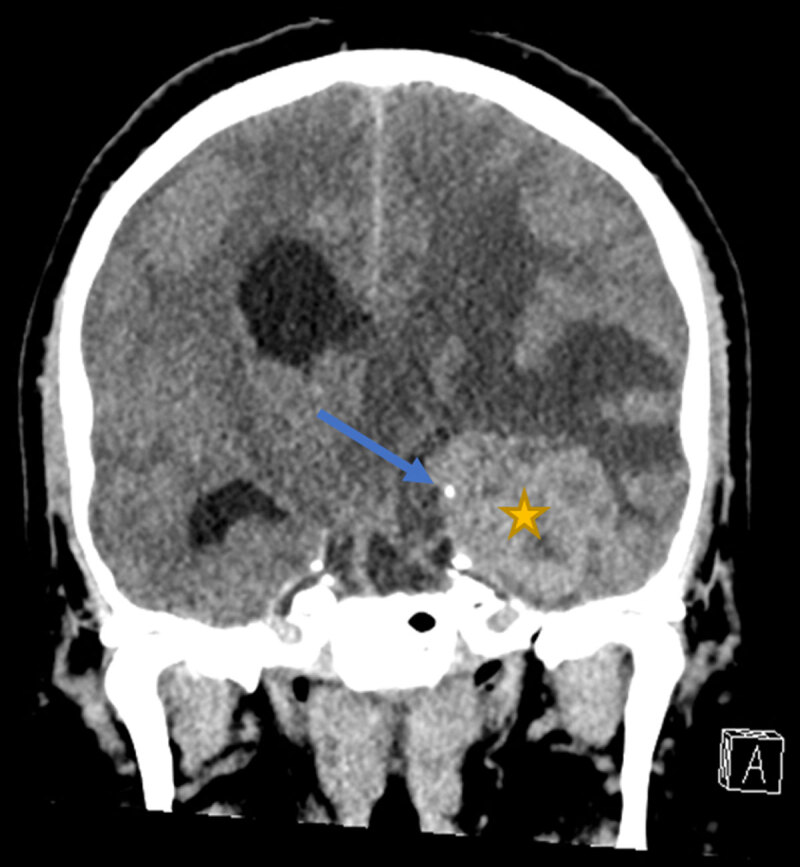


A 49-year-old man presented with gait instability, urinary incontinence, and recurrent headaches for one year. Computed tomography (CT) examination showed a large mass lesion temporo-insular in the left hemisphere (***Figure 1***, star) with perilesional edema, midline shift, and intralesional calcifications (***Figure 1***, blue arrow).

**Figure 2:**
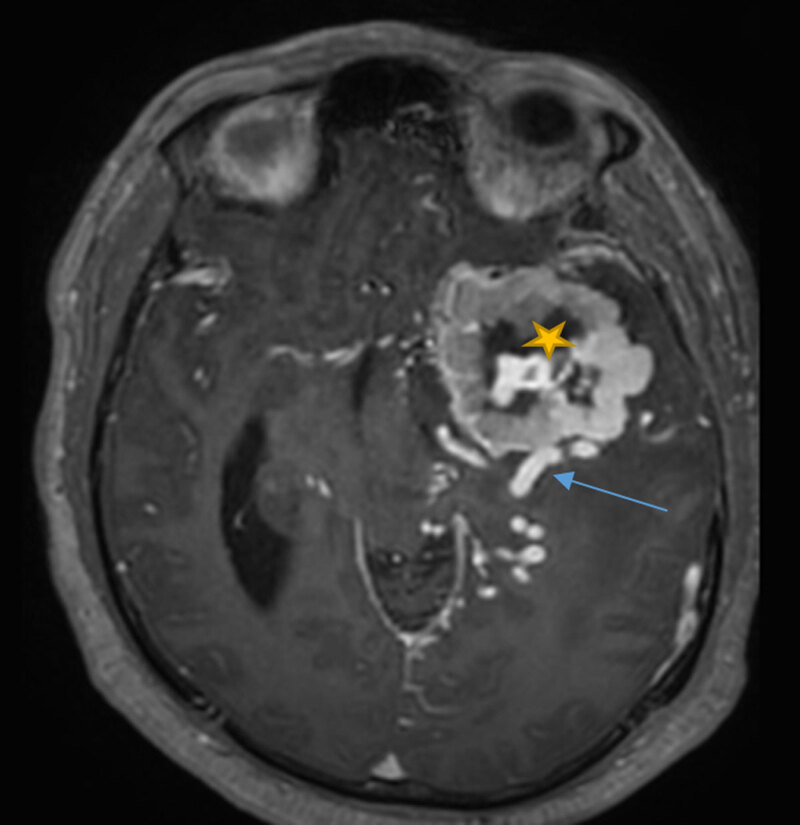


Subsequent magnetic resonance imaging (MRI) confirmed the presence of an extra-axial, dura-based, T2 hyperintense, T1 iso-intense, hypervascular mass (***Figure 2***, star) with large draining veins (***Figure 2***, blue arrow).

**Figure 3:**
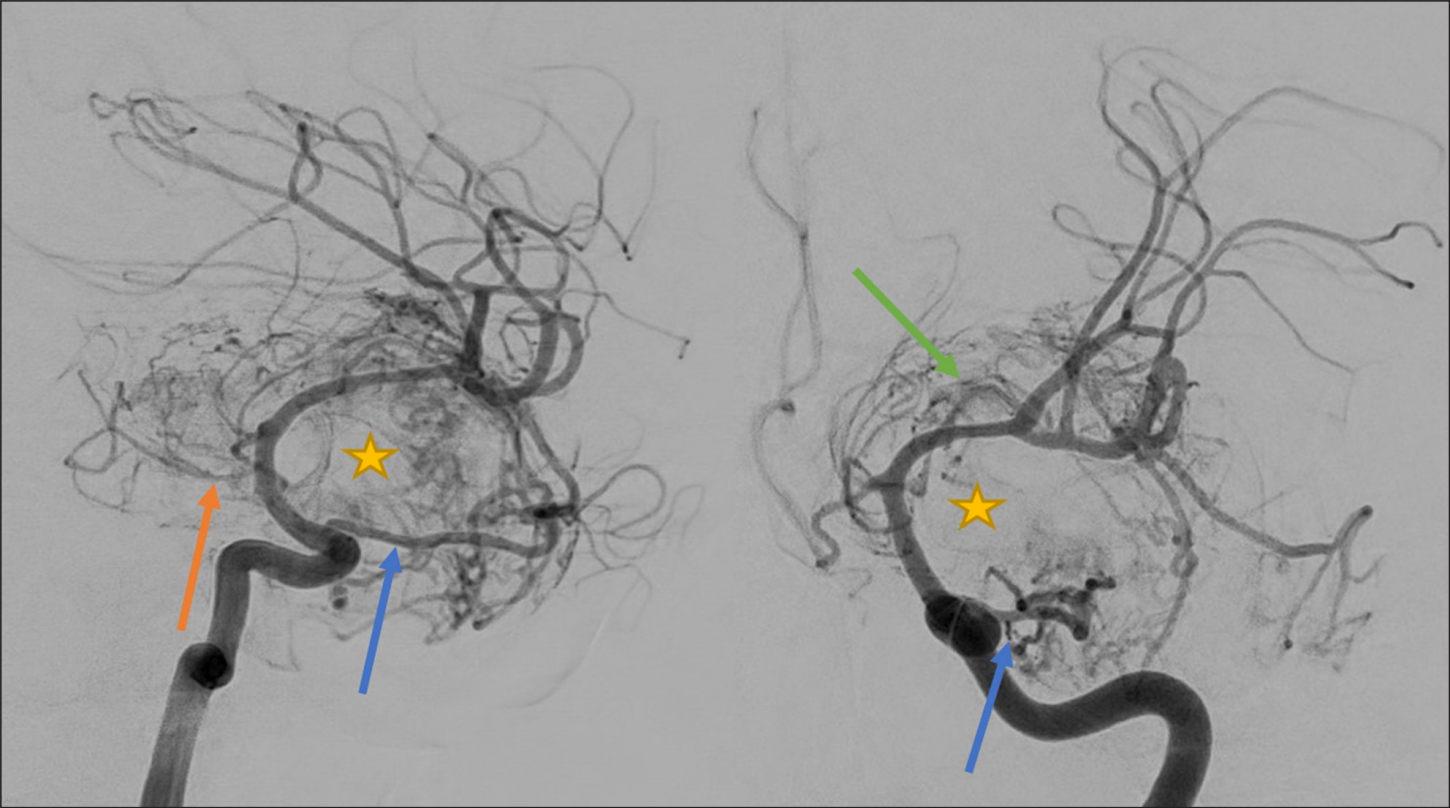


Angiography demonstrated a hypervascular lesion with a mass effect on the middle cerebral artery (***Figure 3***, star). Supplying arteries included the ophthalmic artery (***Figure 3***, blue arrow), anterior choroidal artery (***Figure 3***, orange arrow), and lenticulostriate arteries (***Figure 3***, green arrow), as well as vessels arising from the external carotid, posterior cerebral, and superior cerebellar artery. Because only a very limited part of the tumor was supplied by external carotid braches, embolization was not considered helpful. The differential diagnosis included angiomatous meningioma (AM) and solitary fibrous tumor (SFT).

The patient was treated with surgical resection. Pathology confirmed an AM, WHO grade I.

## Comment

AM is a rare subtype of meningiomas making up around 2% of all meningiomas. AM shows more perilesional edema and flow-voids than other subtypes of meningiomas [1].

SFT, formerly known as hemangiopericytoma, are dura-based lesions with similar imaging features to AM, making the differentiation often challenging. However, compared to AM, SFT show a less favorable prognosis with higher recurrence and metastatic rate.

SFT usually occurs in a younger age group than AM, at an average of 43 years at presentation compared to 65 years for AM.

AM commonly causes hyperostosis, whereas SFT can erode the adjacent bone. Intra-tumoral calcifications are usually absent in SFT. A dural tail sign is typically not present in SFT.

Angiography shows a hypervascular lesion, typically with multiple supplying vessels arising from the external and internal carotid and vertebral arteries. SFT usually does not show early draining veins, which can be seen in AM. Angiography is helpful to assess the possibility of preoperative embolization of AM and SFT.
